# Epidermal bladder cells play a role in water retention in quinoa leaves

**DOI:** 10.5511/plantbiotechnology.24.0807a

**Published:** 2024-12-25

**Authors:** Yasufumi Kobayashi, Yasunari Fujita

**Affiliations:** 1 Biological Resources and Post-harvest Division, Japan International Research Center for Agricultural Sciences (JIRCAS), Tsukuba, Ibaraki 305-8686, Japan; 2 Food Program, JIRCAS, Tsukuba, Ibaraki 305-8686, Japan; 3 Graduate School of Life Environmental Science, University of Tsukuba, Tsukuba, Ibaraki 305-8572, Japan

**Keywords:** drought stress, epidermal bladder cell, quinoa, VIGS, WD40

## Abstract

Quinoa, a pseudocereal and leafy vegetable native to South America, is highly nutritious and can grow in harsh environments. One of the most prominent morphological features of quinoa is that the above-ground portion is covered with a layer of epidermal bladder cells (EBCs), and the role of EBCs in quinoa’s high stress tolerance is of interest. Recent studies have shown that two WD40-repeat proteins, Reduced number of EBC (REBC) and REBC-like1, are required for EBC formation and that EBCs contribute defense mechanisms against biotic stress rather than abiotic stress. However, the role of EBCs in drought stress tolerance remains controversial due to the pleiotropic effects of these genes, including their impact on plant growth. Here, we show that REBC and REBC-like1 mediate water retention in detached quinoa leaves. Using a virus-induced gene silencing (VIGS) system, we found that downregulation of both *REBC* and *REBC-like1* had no apparent effect on plant growth, but reduced the number of EBCs in both lowland and highland quinoa lines. Further, downregulation of both genes increased water loss in detached leaves of quinoa plants, supporting the notion that EBCs mediate water retention in quinoa leaves. Interestingly, we found higher EBC density in the southern highland lines grown in drier areas. Thus, we demonstrate that the effective use of VIGS in the analysis of genes with pleiotropic effects allows analyses that were difficult to perform using mutants alone, and that unlike mutants, functional genomics studies of quinoa can be easily performed in various lines using VIGS.

Climate change is increasing the frequency and severity of various environmental stresses such as drought and salinity around the world, posing a growing threat to crop production. Therefore, it will be necessary not only to confer various environmental stress tolerances to conventional crops, but also to expand the use of orphan crops that already have high environmental stress tolerance but whose use is limited to certain regions (Massawe et al. 2016; Mayes et al. 2012). In this context, quinoa, an orphan crop originally cultivated in the Andean region of South America, has received increasing attention in recent years as a potentially important crop for future food and nutrition security, with the United Nations declaring 2013 the International Year of Quinoa due to its high nutritional value and ability to grow even in harsh environments (Bazile et al. 2016; Kobayashi et al. 2024).

Quinoa, an annual C3 pseudocereal and leafy vegetable of the Amaranthaceae family, which includes spinach (*Spinacia oleracea* L.) and sugar beet (*Beta vulgaris* L.), is an allotetraploid with 2*n*=4*x*=36 chromosomes (Palomino et al. 2008; Ward 2000; Yangquanwei et al. 2013). A most prominent morphological feature of quinoa is that the above-ground surface is covered with a layer of epidermal bladder cells (EBCs), which are modified non-glandular trichomes, also found in the common ice plant; both belong to the order Caryophyllales (Böhm et al. 2018; Shabala and Mackay 2011). EBCs have been intensively studied (Bazihizina et al. 2022; Böhm et al. 2018; Imamura et al. 2020; Kiani-Pouya et al. 2017, 2019; Otterbach et al. 2021), and recent studies have shown that EBCs are involved in herbivore defense mechanisms and not so much in salt stress tolerance (Moog et al. 2022, 2023; Otterbach et al. 2021).

However, the role of EBCs in drought stress tolerance remains controversial. Two WD40-repeat protein (WD40) genes, *Reduced number of EBCs* (*REBC*) (*AUR62039690*) and *REBC-like1* (*AUR62009292*), have been identified as involved in EBC formation in quinoa (Imamura et al. 2020). In the *rebc* mutant, the number of EBCs is reduced (Imamura et al. 2020), and in the *ebcf* mutant, which has mutations in both the *REBC* and *REBC-like1* genes, EBCs are completely absent (Moog et al. 2022). The drought stress tolerance of the *rebc* mutant plants was not different from that of the wild-type (WT) plants (Imamura et al. 2020), whereas the *ebcf* mutant plants were reported to be more drought stress tolerant than the WT plants (Moog et al. 2023). Since the growth of both mutants is significantly delayed compared to WT plants (Imamura et al. 2020; Moog et al. 2023), it would be difficult to make a true comparison of drought stress tolerance from these experiments. On the other hand, both mutants showed aberrant shoot apices after prolonged exposure to wind, in contrast to WT plants (Imamura et al. 2020; Moog et al. 2023), suggesting that these genes and/or EBCs may be involved in some way in drought stress. To gain a better understanding of the role of EBCs in drought stress tolerance, this study examined the effects of reduced EBCs on water retention in quinoa leaves using the virus-induced gene silencing (VIGS) approach to suppress genes to the extent that plant size is not affected.

Seeds of quinoa inbred lines (Iw, J045, J082, J099, and J100) (Mizuno et al. 2020; Ogata et al. 2021) were sown in a peat moss mixture (Jiffy Mix, Sakata Seeds, Yokohama, Japan) in a cell tray and grown in a temperature-controlled phytotron set at 22±2°C under a 12-h light/12-h dark photoperiod. After 7 days, the Iw seedlings were transferred to a standard potting mix (Tsuchitaro, Sumitomo Forestry, Tokyo, Japan) in 0.11-l pots and grown in the temperature-controlled phytotron for 7 days. The J045, J082, J099, and J100 seedlings grown for 10 days after sowing were used for apple latent spherical virus (ALSV) inoculation on the cell tray.

Based on the coding sequence of *REBC* and *REBC-like1* (Moog et al. 2022), we designed PCR primers to amplify 300-bp VIGS trigger regions containing only a single nucleotide mismatch, which could affect both REBC and REBC-like1, in the conserved WD40-repeat domain in *REBC* and *REBC-like1* genes (Figure 1A) as follows; forward; 5′-ATACTCGAGGTCAACCAACAAAGTGGCTACC-3′ and reverse; 5′-TATGGATCCCACCAAATTATCTAGGGTCACTAG-3′ (underlines indicate restriction enzyme sites for XhoI and BamHI, respectively). ALSV-RNA2 vectors for VIGS analyses were constructed as previously described (Ogata et al. 2017, 2021), with minor modifications. The amplified DNA fragments containing trigger sequences from quinoa genes for VIGS were cloned in-frame into the XhoI/BamHI site of pEALSR2 to generate pEALSR2-CqREBC. ALSV inoculations were performed as previously described (Ogata et al. 2021). The plasmids for the ALSV-RNA1 (pEALSR1) and ALSV-RNA2 constructs were mixed in equal amounts, and the DNA solution was mechanically inoculated onto the true leaves of 14-day-old quinoa plants (Iw inbred line) using 600-mesh carborundum (Nacalai Tesque, Kyoto, Japan). The inoculated plants were grown for 2 to 3 weeks and the uninoculated upper leaves showing chlorotic spots were removed. The detached leaves were ground in extraction buffer (0.1 M Tris-HCl, pH 8.0, 0.1 M NaCl, 5 mM MgCl_2_) (Igarashi et al. 2009). Debris was precipitated by centrifugation at 18,800 g for 10 min at 4°C, and the supernatants were used to inoculate quinoa inbred lines grown as described above. Therefore, ALSV derived from quinoa Iw plants inoculated with pEALSR1 and pEALSR2-CqREBC was used as ALSV-REBC, whereas ALSV derived from quinoa Iw plants inoculated with pEALSR1 and pEALSR2 was used as a control (ALSV-WT).

**Figure 1:**
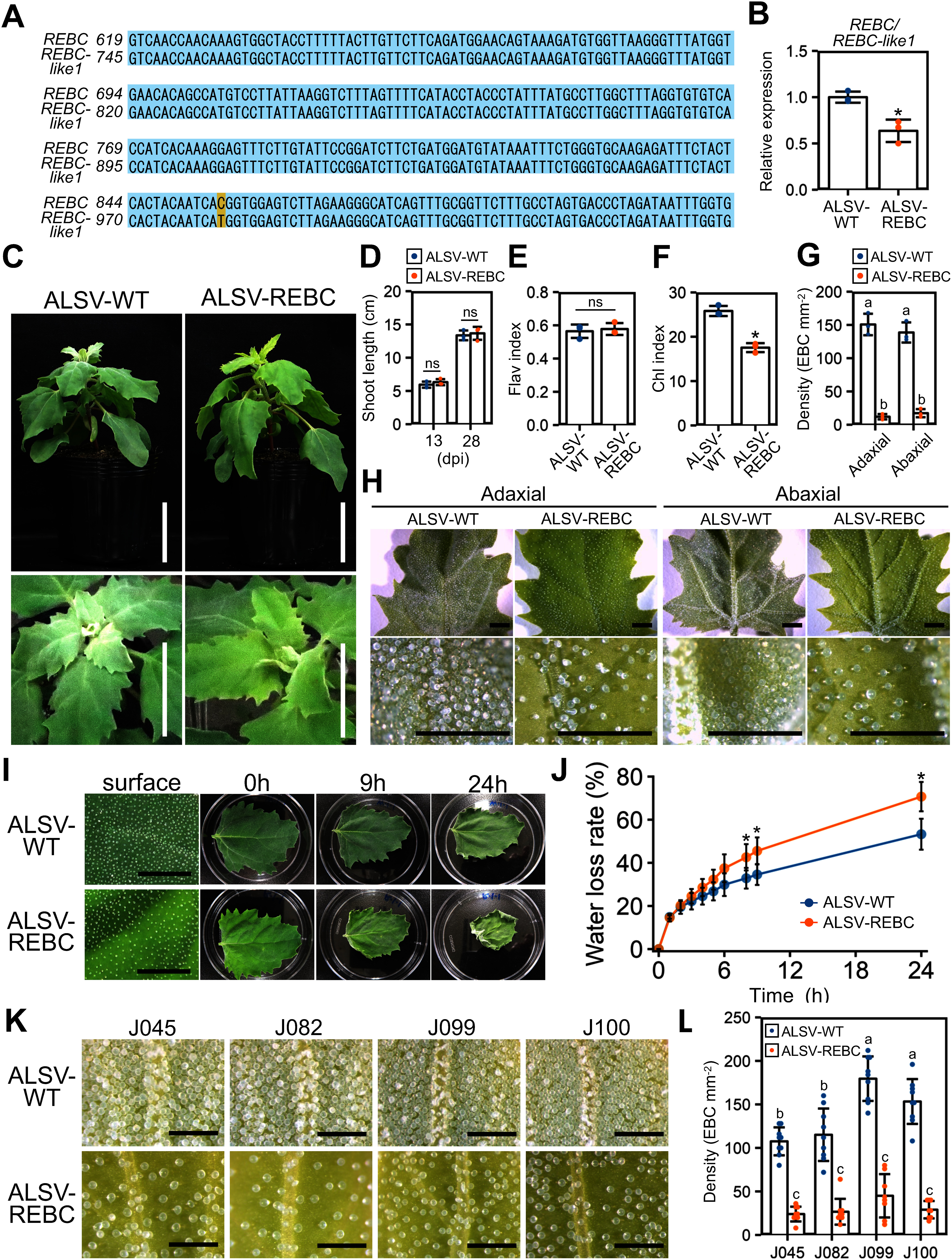
Figure 1. VIGS-mediated downregulation of *REBC* and *REBC-like1* had no apparent effect on plant growth and leaf flavonoid content, but resulted in a slightly paler green color, a significant decrease in chlorophyll content, a marked reduction in the number of EBCs, and a significant increase in water loss in detached leaves of quinoa plants. (A) Two 300-bp VIGS trigger sequences for VIGS in *REBC* and *REBC-like1* genes. The matching and mismatching nucleotides are shown in blue and orange, respectively. (B) RT-PCR quantification of both *REBC* and *REBC-like1* transcripts in the uninoculated upper leaves of plants inoculated with ALSV-REBC and ALSV-WT. Transcript levels of *CqUBQ10* were used as an internal standard. Data were normalized to the ALSV-WT and are presented as means±SD (*n*=3 biologically independent replicates). (C) Growth phenotypes of quinoa Iw inbred lines inoculated with ALSV-REBC and ALSV-WT at 13 dpi. White bars indicate 3 cm. (D) Shoot length of quinoa plants (Iw) inoculated with ALSV-REBC and ALSV-WT at 13 and 28 dpi. Error bars denote SD (*n*=3 biologically independent replicates). (E, F) Epidermal flavonol (E) and chlorophyll (F) contents were measured simultaneously in vivo using a Dualex scientific+ device. Data show means±SD of their contents for each ALSV-REBC and ALSV-WT-inoculated line (*n*=3 biologically independent replicates). (G) EBC densities were calculated for each adaxial and abaxial surface from stereomicroscopy images. Data show means±SD of EBC density for each ALSV-REBC and ALSV-WT-inoculated line (*n*=3 biologically independent replicates). (H) Stereomicroscopy images showing the adaxial (left panels) and abaxial (right panels) surface of the uninoculated upper leaves of the quinoa Iw plants inoculated with ALSV-REBC and ALSV-WT at 21 dpi. Black bars indicate 2 mm. (I) Photographs show changes over time of leaves detached from independent quinoa plants (Iw) inoculated with ALSV-REBC and ALSV-WT and dried on Petri dishes for 0, 9 and 24 h. Black bars indicate 5 mm. (J) Water loss rate of leaves detached from independent quinoa plants (Iw) inoculated with ALSV-REBC and ALSV-WT. Data show means±SD of water loss rates expressed as percent of initial weights (*n*=4 biologically independent replicates). (K) Stereomicroscopy images showing the abaxial surface of the uninoculated upper leaves of quinoa plants inoculated with ALSV-REBC and ALSV-WT at 36 dpi. Four quinoa inbred lines were used: two lowland lines, J045 and J082, and two southern highland lines, J099 and J100. Black bars indicate 1 mm. (L) EBC densities were calculated from stereomicroscopy images. Data show means±SD of EBC density for each ALSV-REBC and ALSV-WT-inoculated quinoa plant (*n*=9 biologically independent replicates). Asterisks and different lowercase letters indicate significant differences at *p*<0.05, as determined using a Student’s *t*-test and one-way analysis of variance with Tukey’s honestly significant difference test. In (H), * *p*<0.05, one-way analysis of variance with Tukey’s honestly significant difference test was used to evaluate differences between the ALSV-REBC-inoculated plants versus the ALSV-WT-inoculated plants at each time point.

Total RNA extraction and reverse transcription (RT)-PCR analysis were performed as previously described (Nagatoshi et al. 2023). The uninoculated upper leaves of the Iw lines inoculated with ALSV-REBC or ALSV-WT were rapidly frozen in liquid nitrogen. RNA was extracted from tissues using RNAiso plus (Takara Bio, Shiga, Japan). First-strand cDNA was synthesized from half a volume of total RNA (1 µg) treated with RQ1 RNase-free DNase (Promega, Madison, WI, United States) using a PrimeScript RT Master Mix (Takara Bio). Gene expression was quantified on a QuantStudio 7 Frex real-time PCR system (Thermo Fisher Scientific, Waltham, MA, United States) using GoTaq qPCR Master Mix (Promega). The primers used in this analysis are described as follows; *REBC*-qRT-F; 5′-ATCTAGCTCCGGCGAAGTCC-3′ and *REBC*-qRT-R; 5′-CCACTGCAAGTTCTCATGTTCC-3′, and *CqUBQ10*-qRT-F; 5′-CCACTCTCCATCTGGTGCTCC-3′ and *CqUBQ10*-qRT-R; 5′-TCCGCAAGAGTCCTTCCATCC-3′ as internal standard.

EBC density was measured on the surface of the uninoculated upper leaves of the quinoa plants inoculated with ALSV-REBC or ALSV-WT at 21 days post-inoculation (dpi). Images were obtained using a Leica MC170 stereomicroscope equipped using a digital camera and a Leica CLS 150X light source. EBCs were counted with NIH ImageJ software (http://imagej.nih.gov/ij/) with the Cell Counter plugin. In addition, leaf pigments (chlorophyll and flavonoid index correlated with their content) were non-destructively assessed using a Dualex scientific+ device (Force-A, Orsay, France), which determines epidermal absorbance in UV-A (wavelength 315–400 nm), mainly due to flavonoids, by comparing chlorophyll fluorescence signals at two different excitation wavelengths (375 and 650 nm) (Cartelat et al. 2005; Goulas et al. 2004). Measurements were performed on the uninoculated upper leaves (fully developed) of the Iw lines at 23 dpi. To determine leaf water loss, leaves of similar size were detached from the Iw lines at 21 dpi. The leaves were weighed immediately, placed on plastic petri dishes in the temperature-controlled phytotron, and weighed hourly for a total of 9 h (except at 7 h time point) and at 24 h. Relative water loss was expressed as a percentage of the initial weight of the leaves, and the initial water loss rate of each leaf was set to 0. All data in this study are presented as mean±standard deviation (SD). Significant differences in data for physiological and morphological indices were determined by one-way analysis of variance and Tukey’s honestly significant difference test or Student’s *t*-test using R version 4.3.0.

To test whether both *REBC* and *REBC-like1* genes affect EBC formation or the other phenotypes, we suppressed the expression of both these genes using VIGS in the quinoa inbred line Iw (Figure 1). RT-PCR analysis showed that endogenous *REBC* expression was significantly downregulated in plants inoculated with ALSV-REBC compared to those inoculated with ALSV-WT (Figure 1B). Downregulation of both *REBC* and *REBC-like1* had no apparent effect on plant growth (Figure 1C, D) and leaf flavonoid content (Figure 1E), but resulted in a slightly paler green color (Figure 1B), a significant decrease in chlorophyll content (Figure 1F), and a marked reduction in the number of EBCs (Figure 1G, H). Notably, VIGS of both *REBC* and *REBC-like1* did not show growth retardation in contrast to their defective mutants (Imamura et al. 2020; Moog et al. 2023). A possible reason for this could be that the level of gene suppression in VIGS was lower than in the previously reported null or deficient mutant. In addition, given the inability of many viruses to infect meristematic and germ cells (Rössner et al. 2022), these results may suggest that there was limited suppression of target gene expression in cells associated with growth suppression in VIGS. The observation that REBC suppression reduces chlorophyll levels is consistent with an earlier report (Imamura et al. 2020), suggesting that *REBC* and *REBC-like1* genes are involved in chlorophyll formation. Suppression of *REBC* and *REBC-like1* gene expression by an average of 36% (Figure 1B) reduced the number of EBCs to an average of 8% in adaxial leaves and 13% in abaxial leaves (Figure 1G), supporting the previous reports that REBC and REBC-like1 mediate EBC formation in quinoa (Imamura et al. 2020; Moog et al. 2022, 2023). Taken together, the VIGS technique successfully produced quinoa leaves with slightly lower chlorophyll content but no growth retardation, but with a significant reduction in the number of EBCs.

Next, we measured water loss in the quinoa leaves obtained by the VIGS technique, which had no significant effect on growth but reduced the number of EBCs, to evaluate their response to drought stress. Downregulation of both *REBC* and *REBC-like1* increased water loss in detached leaves of quinoa plants at all time points (Figure 1I, J). At 24 h after detachment, leaves with downregulated *REBC* and *REBC-like1* had lost 70.7% of their weight compared to when the leaves were detached, whereas leaves from plants inoculated with ALSV-WT had lost 53.3% of their weight compared to when the leaves were detached (Figure 1I, J). These results indicate that REBC and REBC-like1 mediate water retention in detached quinoa leaves. Given that there are no reports that this level of subtle leaf color variation affects water retention of leaves placed under indoor artificial lighting, these data support the notion that EBCs play a role in water retention in quinoa leaves. These results are inconsistent with previous reports using growth retarded mutants, but may be compatible with observations of abnormal shoot apices after prolonged exposure to wind (Imamura et al. 2020; Moog et al. 2023). Considering all these results, it is likely that EBCs are somehow involved in drought stress tolerance, which needs to be further investigated in the future. Thus, we illustrate that the effective use of VIGS in the analysis of genes with pleiotropic effects allows analyses that were difficult to perform using mutants alone.

Both the *rebc* (CQ127, accession PI 614927) and *ebcf* (Titicaca) mutants reported so far in EBC formation, as well as the Iw inbred line used in this study for VIGS analysis, are lowland quinoa lines (Kobayashi et al. 2024; Mizuno et al. 2020; Ogata et al. 2021). However, there is variation in EBC phenotypes among quinoa varieties (Kiani-Pouya et al. 2019). Therefore, we used the ALSV-VIGS system to suppress both *REBC* and *REBC-like1* gene expression in the non-Iw lowland inbred lines J045 (derived from accession Ames13761) and J082 (derived from accession PI614886) and the southern highland inbred lines J099 (derived from accession PI614916) and J100 (derived from accession PI614917). Although the number of EBCs in the leaves of control plants inoculated with ALSV-WT was significantly higher in the southern highland quinoa lines than in the lowland quinoa lines, the number of EBCs in the leaves of plants inoculated with ALSV-REBC was similarly reduced by approximately 70–80% in all lines tested (Figure 1K, L). These results indicate that REBC and REBC-like1 are involved in EBC formation in both southern highland and lowland lines. The higher number of EBCs in the southern highland control lines than in the lowland control lines may be related to the fact that the lowland lines are grown in the temperate region of Chile, while the southern highland quinoa lines are grown in the arid region around the Salar de Uyuni in Bolivia. Future studies should further expand the number of lines examined in terms of *REBC* and *REBC-like1* expression levels, EBC numbers, and growth environment to elucidate the role of EBCs in drought stress. Our research group has laid the groundwork for molecular research on quinoa by sequencing the quinoa genome (Kobayashi et al. 2024; Yasui et al. 2016), performing diversity analyses showing the relationship between genotype and phenotype (Mizuno et al. 2020), and developing the VIGS system to facilitate functional genomics research (Ogata et al. 2021). The data presented here also show that, unlike mutants, functional genomics studies of quinoa can be easily performed in a variety of lines using VIGS technology.

## Abbreviations

ALSV: apple latent spherical virus
EBC: epidermal bladder cell
*ebcf*: *ebc-free*
REBC: reduced numbers of EBCs
VIGS: virus-induced gene silencing
WD40: WD40-repeat protein
WT: wild-type
